# Effects of Methyl Substitution and Leaving Group on E2/S_N_2 Competition for Reactions of F^−^ with RY (R = CH_3_, C_2_H_5_, ^i^C_3_H_7_, ^t^C_4_H_9_; Y = Cl, I)

**DOI:** 10.3390/molecules28176269

**Published:** 2023-08-27

**Authors:** Wenqing Zhen, Siwei Zhao, Gang Fu, Hongyi Wang, Jianmin Sun, Li Yang, Jiaxu Zhang

**Affiliations:** State Key Laboratory of Urban Water Resource and Environment, School of Chemistry and Chemical Engineering, Harbin Institute of Technology, Harbin 150001, China; 20b925102@stu.hit.edu.cn (W.Z.); 18b925114@stu.hit.edu.cn (S.Z.); 20b925088@stu.hit.edu.cn (G.F.); wanghongyi999123@163.com (H.W.); sunjm@hit.edu.cn (J.S.)

**Keywords:** base-induced elimination (E2), nucleophilic substitution (S_N_2), α-methyl substitution, electronic structure calculation

## Abstract

The competition between base-induced elimination (E2) and bimolecular nucleophilic substitution (S_N_2) is of significant importance in organic chemistry and is influenced by many factors. The electronic structure calculations for the gas-phase reactions of F^−^ + RY (R = CH_3_, C_2_H_5_, ^i^C_3_H_7_, ^t^C_4_H_9_, and Y = Cl, I) are executed at the MP2 level with aug-cc-pVDZ or ECP/d basis set to investigate the α-methyl substitution effect. The variation in barrier height, reaction enthalpy, and competition of S_N_2/E2 as a function of methyl-substitution and leaving group ability has been emphasized. And the nature of these rules has been explored. As the degree of methyl substitution on α-carbon increases, the E2 channel becomes more competitive and dominant with R varying from C_2_H_5_, ^i^C_3_H_7_, to ^t^C_4_H_9_. Energy decomposition analysis offers new insights into the competition between E2 and S_N_2 processes, which suggests that the drop in interaction energy with an increasing degree of substitution cannot compensate for the rapid growth of preparation energy, leading to a rapid increase in the S_N_2 energy barrier. By altering the leaving group from Cl to I, the barriers of both S_N_2 and E2 monotonically decrease, and, with the increased number of substituents, they reduce more dramatically, which is attributed to the looser transition state structures with the stronger leaving group ability. Interestingly, ∆*E*_0_^‡^ exhibits a positive linear correlation with reaction enthalpy (∆*H*) and halogen electronegativity. With the added number of substituents, the differences in ∆*E*_0_^‡^ and ∆*H* between Y = Cl and I likewise exhibit good linearity.

## 1. Introduction

Two basic organic reactions in the development of modern physical organic chemistry, base-induced bimolecular elimination (E2) and bimolecular nucleophilic substitution (S_N_2) reactions, usually compete with each other in many cases. In the past few decades, E2 and S_N_2 reactions have been widely researched in the gas and condensed phase, both experimentally [1,2,3] and theoretically [4,5,6,7,8,9,10,11,12,13,14,15,16,17,18,19]. Since S_N_2 and E2 pathways generate the same ionic product, it is a serious challenge to distinguish between the two processes using conventional spectrometric techniques [20]. Kinetic isotope effects could qualitatively tell E2 and S_N_2 pathways apart, as it is observed that E2 reactions have normal KIEs (k_H_/k_D_ > 1), whereas S_N_2 reactions have inverse KIEs (k_H_/k_D_ < 1) [1]. In this regard, theoretical approaches have played an important role in probing the mechanisms. Extensive theoretical investigations have mainly focused on the reactions of the type of X^−^ + CH_3_CH_2_Y (X = F, Cl, OH, ClO, et al.; Y = F, Cl, Br, I) over the years [13,14,16,17,21,22,23,24,25,26,27,28,29]. The competition between E2 and S_N_2 reaction pathways caused by multiple factors, such as nucleophile, leaving groups, substrate characteristics, and environment, has been investigated in detail [10,16,17,25,28,29,30,31]. Mugnai et al. [10] analyzed the influence of temperature on the competition between E2 and S_N_2 in F^−^ + CH_3_CH_2_Cl reaction by ab initio molecular dynamics, and found that S_N_2 and E2 reaction mechanisms were favored at high and low temperatures, respectively. In addition, the ion-imaging experiments and quasi-classical trajectory simulations of this system conducted by Wester et al. [31] also showed that the S_N_2 mechanism becomes more relevant as the collision energy is increased. Bickelhaupt and colleagues [22], founded on an activation-strain analysis, reported that F^−^ could participate in a more stabilizing orbital interaction with CH_3_CH_2_Cl than PH_2_^−^, owing to its augmented proton affinity, leading to the observed preference for E2. Shaik et al. [24]. from the VB perspective, revealed that essentially S_N_2 was the preferred reaction pathway but the E2 pathway prevailed in many cases on account of the greater resonance stabilization in its transition-state (TS) region.

Among these factors, substituent effects have revealed an important function in studying the mechanisms and the competition between the E2 and S_N_2 [32,33,34,35,36,37]. Gronert [28,29] measured fluoride reacting with CH_3_Cl with a reaction efficiency of 0.56, and with the increased degree of substitution to ethyl, isopropyl, and tert-butyl chloride, the efficiency improved from 0.79 to 0.93 due to the presence of an E2 pathway, which was entropically favored [36]. Further theoretical calculations at the MP2/6-31 + G** level for the reaction of F^−^ with CH_3_CHClCH_3_ pointed out methyl substitution at the α-carbon elevated the barrier of the S_N_2 pathway due to the crowded environment while stabilizing the transition state in E2 reactions [38]. Accordingly, Bierbaum et al. [1] explored the competition between E2 and S_N_2 pathways for BrO^−^ and ClO^−^ with RCl (R = CH_3_, C_2_H_5_, ^i^C_3_H_7_, ^t^C_4_H_9_) by using deuterium kinetic isotope effect (KIE) means. For each anion series, the trend that KIEs became increasingly more normal (k_H_/k_D_ > 1) as the extent of substitution indicated that the E2 pathway became the predominant pathway and steric effects inhibited the S_N_2 pathway. A method calculating “steric hindrance (SH)” was proposed by Pendás and co-workers [39,40], which was consistent with the chemical intuition that the SH of the complex methylated alkyl system S_N_2 was greater than E2.

**Scheme 1:**
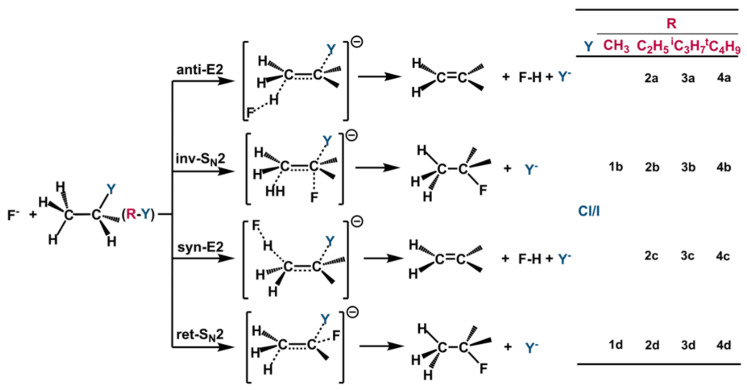
Pathways for reactants F^−^ + RY (R= CH_3_, C_2_H_5_, ^i^C_3_H_7_, ^t^C_4_H_9_,represented in red; Y = Cl, I, represented in blue).

Recently, Wester et al. [41] presented a series of reactions X^−^ + RY (X = F, Cl, R = CH_3_, C_2_H_5_, ^i^C_3_H_7_, ^t^C_4_H_9_ and Y = Cl, I), which displayed the transition from backward to forward scattering by the substitution of the α-carbon. It is noticed that the changes following increasing methyl substitution are somewhat different for different leaving groups. Compared to the rich experimental studies, systematic and accurate PESs for complex methylated alkyl halides, especially ^i^C_3_H_7_Y and ^t^C_4_H_9_Y, are still lacking. In this paper, a series of F^−^ + RY (R = CH_3_, C_2_H_5_, ^i^C_3_H_7_, ^t^C_4_H_9_; Y = Cl, I) reactions as shown in Scheme 1 have been investigated with electronic structure calculations. We revisit these systems and try to address the following two issues. (1) The accurate PESs for a series of reactions are established and compared to explore how methyl substitution at the α-carbon affects the E2/S_N_2 competing mechanisms. (2) It will be of interest to probe the role of the leaving group in E2/S_N_2 reactions of α-methyl substitution.

## 2. Results and Discussion

### 2.1. Potential Energy Surfaces of F^−^ + RY Reactions

**Figure 1:**
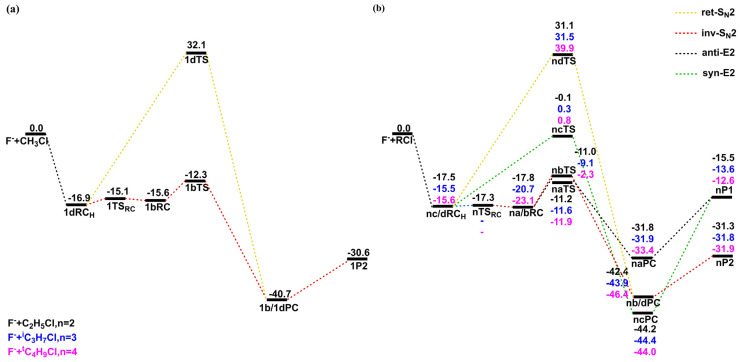
Potential energy curves and stationary points at MP2/aug-cc-pVDZ level for F^−^ + RCl reactions. The energy (in kcal/mol) is relative to F^−^ + CH_3_Cl (**a**) and F^−^ + RCl (**b**) reactants at 0 K, and does not include ZPE. In (**b**), the black, blue, and pink numbers represent F^−^ + C_2_H_5_Cl, F^−^ + ^i^C_3_H_7_Cl, and F^−^ + ^t^C_4_H_9_Cl reactions, respectively.

The relative energy of stationary points of F^−^ + RCl (R = CH_3_, C_2_H_5_, ^i^C_3_H_7_, ^t^C_4_H_9_ and Y = Cl, I) calculated by the MP2 method are displayed in Table 1 together with the high-level CCSD(T)-F12b [16,42], CCSD(T) [17] benchmark values, and the available B97-1/aug-cc-pVDZ values [43]. The average deviation from the benchmark is 0.11/0.48, 0.16/0.13, and 0.16/0.35 kcal/mol for CH_3_Cl/I, C_2_H_5_Cl/I, and ^i^C_3_H_7_Cl/I, respectively, indicating that the MP2/aug-cc-pVDZ (ECP/d) level is reasonable. The profiles of PES for F^−^ + RY are presented in detail in Figure 1a,b and Figure S1a,b together with the geometrical structures in Figure S2a,d. As shown in Figure 1 and Figure S1, similar to the reaction of F^−^ with ethyl halide, for isopropyl and tert-butyl reactions, four traditional reaction pathways, including base-induced anti and syn elimination (anti-E2 and syn-E2), as well as nucleophilic substitution with inversion and retention of configuration (inv-S_N_2 and ret-S_N_2), are predicted by MP2 theory. Inv-S_N_2 and anti-E2 reactions share the same reactant complex (a/bRC), and ret-S_N_2 and syn-E2 also utilize a common complex (c/dRC) along the reaction coordinate. With the increased methyl substitution, the difference in PES profiles of the series of reactions lies in the entrance channel. For F^−^ + RY (R = CH_3_, C_2_H_5_, and Y = Cl, I), a hydrogen-bonded F···HC^α^H_2_(CH_2_)Y complex (c/dRC_H_) is obtained, which can easily convert to an ion-dipole complex F···H^β^CH_2_(CH_2_)Y (a/bRC) via a low-energy TS_RC_ [16,29,42,44,45,46,47,48,49,50,51,52]. In contrast, for F^−^ + RCl (R = ^i^C_3_H_7_, ^t^C_4_H_9_ and Y = Cl, I) reactions, only an ion-dipole complex (a/bRC) is found, where F^−^ is situated between α-carbon and β-hydrogen of iso-propyl or tert-butyl moiety, so that F^−^ can attack either target atom to form an inv-S_N_2 or anti-E2 channel. For the reactant complex F···H^β^H_2_(CH_3_)CHY (c/dRC) in syn-E2 and ret-S_N_2 channels, F^−^ and some hydrogen atoms in β-position, instead of α-hydrogen of alkyl halides, have a mild hydrogen-bond interaction. There is no transition state available for the conversion of these two RCs.

**Figure 2:**
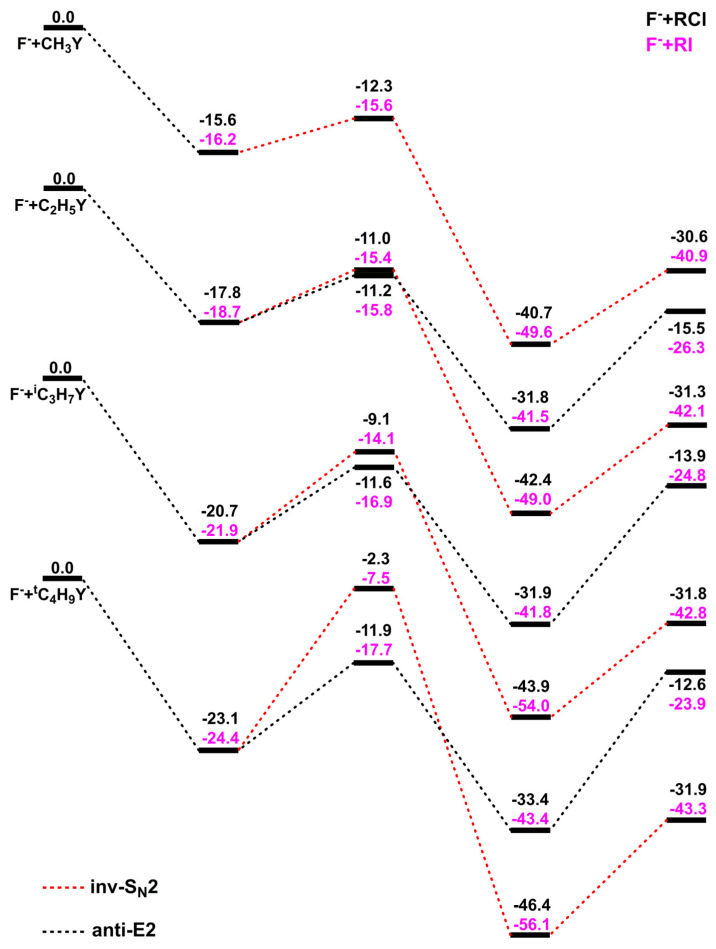
Schematic potential energy surface (PES) of the F^−^ + RY (R = CH_3_, C_2_H_5_, ^i^C_3_H_7_, ^t^C_4_H_9_; Y = Cl, I) with stationary points along the anti-E2 (black) and inv-S_N_2 (red) channels at MP2/aug-cc-pVDZ(ECP/d) level. The reported energies (in kcal/mol) are relative to reactants without ZPE.

Resulting from the strong steric exclusion between the nucleophile and substrate, the ret- S_N_2 TS usually gives a much higher overall barrier than other reaction pathways, suggesting it is the least favorable pathway. The barrier for the syn-E2 pathway is usually higher than inv-S_N_2 and anti-E2 for a similar hindrance effect. Therefore, in the following discussions, the most competitive inv-S_N_2 and anti-E2 pathways for the series of F^−^ + RY reactions are considered here, and their PES profiles obtained at the MP2 level of theory are characterized in Figure 2 for convenience of comparison. For the F^−^ + CH_3_Y reaction, F^−^ attacks CH_3_Y on the back-side via a traditional path along a pre-reaction ion-dipole complex (1bRC), a Walden-inversion transition state (1bTS), and a post-reaction ion-dipole complex FCH_3_···Y^−^ (1bPC). The E2 pathways appear with the successive addition of the methyl group besides the S_N_2 pathways and show similar double well potential characters. The initial association of F^−^ and RY can form an ion-dipole complex a/bRC and, after going over a/bTS, the system drops down to the deep potential energy well a/bPC and then decomposes to products P1 (RF + Cl^−^/I^−^) and P2 (RCH_2_ = CH_2_ + HF + Cl^−^/I^−^), respectively.

### 2.2. Effects of α-Methyl Substitution 

**Scheme 2:**
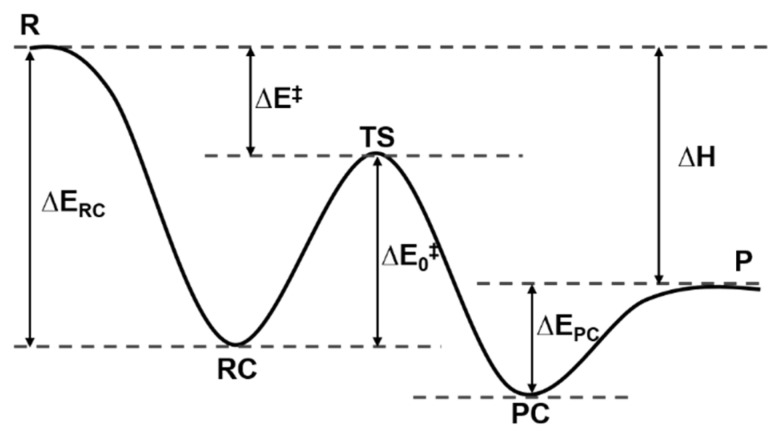
Schematic of activation barrier (∆*E*_0_^‡^), overall barrier (∆*E*^‡^), and reaction enthalpy ∆*H*.

To explore the effects of the addition of the α-methyl group on the competition of E2 and S_N_2 mechanisms, the activation (∆*E*_0_^‡^) and the overall barrier (∆*E*^‡^) are especially emphasized for discussion as presented in Scheme 2, and the calculated values of the relevant energies are summarized in Table 1. Here, Y = Cl is used as an example.

Exothermicity. As shown in Figure 2, it is clear that all E2 and S_N_2 paths for the reaction of F^−^ + RY (R = CH_3_, C_2_H_5_, ^i^C_3_H_7_, ^t^C_4_H_9_, and Y = Cl, I) are highly exothermic, and, for each reaction, the reaction enthalpy ∆*H* of S_N_2 pathways is much more negative than that of E2 pathways, suggesting S_N_2 reactions are more exothermic than E2 reactions owing to the strong combination between the F atom and C atoms in the neutral products. By changing R from the methyl to the tert-butyl group, the ∆*H* of S_N_2 pathways slightly drops, while the values of E2 pathways escalate, eventually widening the ∆*H* gap between E2 and S_N_2.

**Figure 3:**
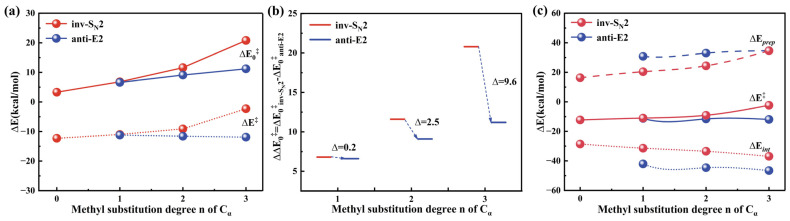
(**a**) Activation barrier heights ∆*E*_0_^‡^ (full line) and overall barrier heights (dashed line) of anti-E2 and inv-S_N_2 transition states as a function of methyl substitution degree *n* of C^α^ (*n* = 0–3). (**b**) Barrier difference ∆∆*E*_0_^‡^ between anti-E2 and inv-S_N_2 as a function of *n* (*n* = 1–3). Values of Y = Cl are reported. All the energies are in kcal/mol. (**c**) Energy decomposition analysis of the anti-E2 and inv-S_N_2 transition structure between F^−^ + (CH_3_)_n_CCl (*n* = 0–3).

Barrier Height. The variation of ∆*E*_0_^‡^, ∆*E*^‡^, and the difference in ∆*E*_0_^‡^ between inv-S_N_2 and anti-E2 along the methylation of the α-carbon is illustrated in Figure 3a–c. The horizontal coordinate is the degree of methyl substitution of C^α^, named *n*, ranging from 0 to 3. As described in Figure 3a, successively adding methyl groups to the C^α^ dramatically raises the activation barrier (∆*E*_0_^‡^) of inv-S_N_2 from 3.3 to 6.8 to 11.6 and finally to 20.8 kcal/mol, while the ∆*E*_0_^‡^ of anti-E2 gently escalates from 6.6, 9.1 to 11.2 kcal/mol. Figure 3b depicts the activation barrier difference between the inv-S_N_2 and anti-E2 pathways (∆∆*E*_0_^‡^ = ∆*E*_0_^‡^_inv-SN2_ − ∆*E*_0_^‡^_anti-E2_) as *n* changes from 1 to 3, which is 0.2 and 2.5 kcal/mol for *n* = 1 and 2, respectively. The significant augment of ∆∆*E*_0_^‡^ for the F^−^ + ^t^C_4_H_9_Cl reaction is observed in doubling the difference to 9.6 kcal/mol. All these results suggest that anti-E2 is becoming more and more competitive. Wester and co-workers [32] have disentangled the dynamics of the competition between anti-E2 and inv-S_N_2 in the reaction F^−^ + C_2_H_5_Cl, indicating that anti-E2 is more advantageous. In addition, Gronert [38] also predicted that in the reaction of F^−^ + ^i^C_3_H_7_Cl anti-E2 is completely dominated and substitution should be more competitive with ethyl halides. As the maximum difference between ∆*E*_0_^‡^(S_N_2) and ∆*E*_0_^‡^(anti-E2) of these three systems, the anti-E2 mechanism will also be the most favorable pathway for F^−^ + ^t^C_4_H_9_Cl (I).

For the variation pattern of overall barriers (∆*E*^‡^), the α-methyl group slightly changes the ∆*E*^‡^ of an anti-E2 pathway with values gradually dropping from −11.2, −11.6 to −11.9 kcal/mol but significantly alters the barrier of inv-S_N_2 from −12.3 kcal/mol for F^−^ + CH_3_Cl to −2.3 kcal/mol for F^−^ + ^t^C_4_H_9_Cl. Rablen et al. [35] systematically compared the free energies of S_N_2/E2 transition states for CN^−^ + RCl reactions at the W1 and G4 levels of electronic structure theory in the presence of a simulated acetonitrile solvent. Their results suggest that the barrier to the E2 reaction reduces by the same magnitude as the barrier to S_N_2 raises when methylation of the α-carbon increases. Connor and Gronert [34] studied the impact of α- and β-methylation on E2 and S_N_2 reactions between a series of alkyl bromides and nucleophiles in the gas phase using both mass spectrometer experiment and computational methods. They found the reduction in S_N_2 rate constant and the mounting in E2 rate constant when adding a methyl group to the α-carbon position, which is in line with our findings.

To understand why the increasing alkyl substitution strikingly enhances the ∆*E*^‡^ of the S_N_2 reaction and slightly lowers the E2 barrier, we decompose the transition state energy by referring to the activation strain analyses proposed by Bickelhaupt et al. [28,53,54] as shown in Figure 3b. Energy decomposition contributes to a quantitative understanding of how methyl substitution affects the inv-S_N_2/anti-E2 reaction barrier. The total energy ∆*E*^‡^, that is, the difference between reactants and transition states, can be decomposed into preparation energy (∆*E*_prep_) and interaction energy (∆*E*_int_) based on the formula ∆*E*^‡^ = ∆*E*_prep_ + ∆*E*_int_. The ∆*E*_prep_ is the energy that is needed to overcome the deformation of individual reactants from their equilibrium structure into the geometries of the transition structure. And the interaction energy ∆*E*_int_ is considered as the energy difference between the individual fragments of transition states’ geometries and the transition states. For F^−^ + RCl reactions, the inv-S_N_2 goes with less preparation energy than anti-E2, which can be attributed to the different mechanisms. One bond breaking occurs in the inv-S_N_2 mechanism, whereas two bond breakings and one C-C bond shrinking occur along the anti-E2 pathway. Hence, the destabilizing distortion characteristic for the anti-E2 reaction pathway is by definition higher than the inv-S_N_2 reaction pathway. As previously proposed by Bickelhaupt [53], the C^α^-Y bond extension of inv-S_N_2 reduces the antibonding orbital overlap between C_2p_ and Y_2p_ orbitals, which makes the LUMO of the substrate more stable. Obviously, due to the antibonding orbital overlap of both the C^α^-Y and C^β^-H bonds being diminished, this stabilization of the LUMO is more significant in the E2 reaction. For inv-S_N_2 transition structures, when the degree of CH_3_ varies from 0 to 3, ∆*E*_prep_ increases gradually, indicating the fragments in the transition structures distorted more violently, whereas ∆*E*_int_ decreases, suggesting the interaction of two parts is stronger. The decline in ∆*E*_int_ cannot pay for the rapid growth of ∆*E*_prep_, resulting in the overall rise in ∆*E*^‡^. Especially in the F^−^ + ^t^C_4_H_9_Cl reaction, the increase in S_N_2 ∆*E*^‡^ is particularly significant. In comparison, the ∆*E*_prep_ of the anti-E2 transition structure is higher but elevates more slowly than that of the inv-S_N_2. Accordingly, the results obtained suggest that, compared to the anti-E2 reaction, the inv-S_N_2 reaction is more sensitive to structural changes in the substrate. Therefore, the barrier of inv-S_N_2 increases with the increased degree of substitution, resulting in less competitiveness compared to anti-E2, which is in agreement with previous research by Pendás et al. [39]. The ∆*E*_int_ is further broken down into the steric term ∆*E*_steric_ (∆*E*_steric_ = ∆*E*_els_ + ∆*E*_XC_ + ∆*E*_Pauli_) and the orbital interaction term ∆*E*_orb_ in order to ascertain the primary contributor to the fluctuation in ∆*E*_int_ as shown in Figure S3 [55]. Obviously, the orbital interaction term ∆*E*_orb_ is responsible for the interaction energy. The orbital interactions of both S_N_2 and E2 decrease as the number of substituents increases. It is noteworthy that the stronger orbital interaction favors the E2 reaction to lower ∆*E*_int_ compared to its S_N_2 analog. This is consistent with the finding by Bickelhaupt et al. [22] for the F^−^ + CH_3_CH_2_Cl reaction.

These results suggest that, with the addition of α-methyl substituent, the anti-E2 reaction is completely dominant considering the energetics from ethyl to butyl reactions in the gas phase. This is consistent with the scattering experimental phenomenon [41] that the character of forward scattering becomes more and more obvious with the increased size of residue R, where scattering into the forward hemisphere is a mechanistic fingerprint of E2 reactions. Further dynamics simulations are desired for revealing the variation in an aspect of dynamical factors.

### 2.3. Effects of Leaving Group 

**Figure 4:**
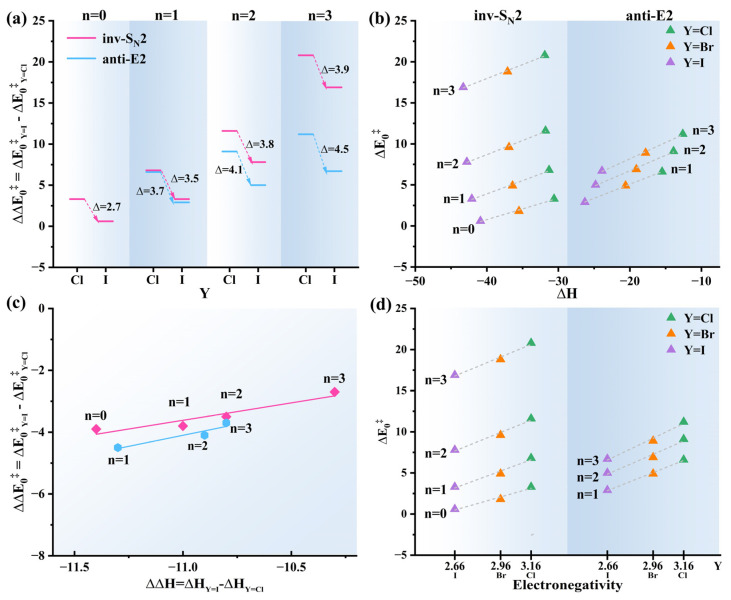
(**a**) Comparison of activation barriers between inv-S_N_2 and anti-E2 TS in the F^−^+ RY reaction calculated by MP2 method. The correlation of (**b**) activation barrier heights ∆*E*_0_^‡^ and reaction enthalpy ∆*H*, (**c**) activation barrier difference ∆∆*E*_0_^‡^ and reaction enthalpy ∆∆*H* between Y = Cl and I, and (**d**) activation barrier heights and electronegativity of leaving group Y.

It is of interest to explore the effect of the leaving group along the α-methyl substitution. Figure 4a compares the activation barrier heights of F^−^ + RCl and F^−^ + RI; it can be seen that, as the enhanced leaving group ability varies from Cl to I, both S_N_2 and E2 reaction barriers are dropped by similar amounts. More significantly, with the increased number of substituents of *n* = 0–3 the barrier heights decrease more dramatically from 3.5/3.7, 3.8/4.1, to 3.9/4.5 kcal/mol for the S_N_2/E2 reaction pathways along Y = Cl to I. Their key TS structural character could be closely related to this change. Variation in the leaving group from Cl to I causes the elongation of the C^α^-Y bond ∆L (L_Cα-I_ − L_Cα-Cl_) ranging from 0.26, 0.29, 0.32 to 0.38Å with *n* going from 0 to 3 for the TS structure of inv-S_N_2. Similarly, the bond elongation ∆L of C^α^-Y for anti-E2 TS also grows along with methyl substitution degree *n*. The looser transition states typically align with the reduced barrier heights, resulting in heightened reactivity [20,56,57]. 

Along the leaving group ability changing from Cl, Br to I, a good linear relationship is found between ∆*E*_0_^‡^ and ∆*H* for both inv-S_N_2 and anti-E2 pathways of each F^−^ + RY reaction as presented in Figure 4b. Obviously, the F^−^ + RY reaction set obeys the expression ∆*E*_0_^‡^ = a∆*H* + C, which connects the barrier with the reaction enthalpy, following the Bell–Evans–Polanyi principle, which is a long-standing chemical theory [58]. Furthermore, good relevance is also found between activation barrier difference ∆∆*E*_0_^‡^ and enthalpy difference ∆∆*H* for Y = Cl and I with the increased degree of substitution of the corresponding S_N_2 and E2 pathways as shown in Figure 4c. The above results indicate that, as the leaving group changes from Cl to I, the reaction becomes more exothermic and the barrier drops. Barriers of both inv-S_N_2 and anti-E2 reactions are found to exhibit good linear dependences with halogen electronegativity increasing in the order of I (2.66) < Br (2.96) < Cl (3.16) as shown in Figure 4d. The above results suggest the decreased electronegativity of Y is expected to lead to a looser TS structure (elongation of C-Y bond), and further a higher reaction reactivity.

## 3. Computational Methods

The stationary points for a series of F^−^ + RY (R = CH_3_, C_2_H_5_, ^i^C_3_H_7_, ^t^C_4_H_9_ and Y = Cl, I) reactions are studied by second-order Møller–Plesset perturbation MP2 [59,60] with the frozen core (FC) method. The atoms of H, C, F, and Cl are based on Dunning and Woons aug-cc-pVDZ [61,62] basis set. For I, the core electrons use the Wadt and Hay ECP [63] and the valence electrons use a 3s, 3p basis, plus a d-polarization function with a 0.262 exponent, and s, p, and diffuse functions with exponents of 0.034, 0.039, and 0.0873, respectively. 

According to previous work, aug-cc-pVDZ basis set has the lowest systematic errors, while G** and aug-cc-pVTZ tend to overestimate and underestimate the single-point energies [64]. Aug-cc-pVDZ basis set also showed good agreement with the experiment in previous research on similar reactions [17,30,42]. Vibrational analysis is used to determine each stationary point under the harmonic oscillator mode in which balanced structures have no imaginary frequencies and transition states have one normal mode with an imaginary frequency. Furthermore, each transition state is calculated with the internal reaction coordinate (IRC) to make sure that it connects the assumed pre- and post-reaction complexes. The coupled cluster theory with triple excitations treated perturbatively CCSD(T) is often used as a benchmark due to its good accuracy [16,17,25,65]. Hence, the relative energies of the stationary points are compared with the high-level CCSD(T)-F12b [16,42] and CCSD(T) [17] benchmark values, and the available B97-1 values [43] for methyl, ethyl, and tert-butyl halide systems, to rationalize the dependability of the computing method. Since there is no report available previously for isopropyl halide reactions, the high-level CCSD(T)/aug-cc-pVTZ or CCSD(T)/ECP/d energy corrections based on MP2 geometries are performed. Gaussian 09 package [66] was used for all computations.

## 4. Conclusions

In this paper, F^−^ ions with a series of α-substituted alkyl chlorides and alkyl iodides in the gas phase are studied by using MP2/aug-cc-pVDZ or MP2/ECP/d methods, and the effect of methyl substituents and leaving group ability on the competition of E2 and S_N_2 pathways is investigated. As the degree of methyl substitution increases, the preference for anti-E2 over inv-S_N_2 enlarges, and, till R = ^t^C_4_H_9_, anti-E2 becomes overwhelmingly dominant. The prediction of this reaction trend is consistent with the differential scattering experiment [41]. This can be explained by energy decomposition analysis. With the increased degree of substitution, the drop in the interaction energy between reactants cannot compensate for the rapid growth of preparation energy resulting from the more distorted transition state structure for the inv-S_N_2 reaction pathway.

In the aspect of the leaving group, the barrier heights for both E2 and S_N_2 reactions drop more dramatically along Y = Cl to I with the increased number of substituents, which can be attributed to the relaxation of the key transition state structure. Variation in the leaving group from Cl to I results in the larger extension of the C^α^-Y for the TS structure of anti-E2 and inv-S_N_2 with the methyl group going from 0 to 3, and thus the larger reaction activity. Along the leaving group ability changing from Cl, Br to I, a notable linear relationship is found between ∆*E*_0_^‡^ and ∆*H* for both the inv-S_N_2 and anti-E2 channels of each F^−^ + RY reaction, indicating the smaller the reaction barrier, the more negative the reaction enthalpy. These results correspond to the decreasing halogen electronegativity from Cl to I, which results in the more relaxed structural characters of TS and thus the larger reaction probability. This work lays the foundation for our subsequent dynamic simulations. Since it is known that the dynamics of the reaction may deviate from the potential energy surface, dynamics simulations are able to intensify our understanding of the effects of the substitution and leaving group on the competition mechanism between E2 and S_N_2 reactions.

## Figures and Tables

**Table 1:** The relative energies (kcal/mol) for stationary points of the E2 and S_N_2 pathways on the F^−^ + RY with different methods.

		Cl			I		
R	Species	MP2	CCSD(T) ^a^	Exptl ^c^	MP2	CCSD(T) ^b^	Exptl ^c^
CH_3_	1dRC_H_	16.9	17.6		−18.9	−20.3	
	1TS_RC_	−15.1	−15.8		−15.8	−17.6	
	1bRC	−15.6	−16.1		−16.2	−18.3	
	1bTS	−12.3	−12.8		−15.6	−18.1	
	1dTS	32.1	30.7		22.8	18.3	
	1dPC	−40.7	−41.6		−49.6	−55.3	
	1bPC	−40.7	−41.6		−49.6	−55.3	
	1P2	−30.6	−31.8	−31.1	−40.9	−48.0	−42.3
C_2_H_5_	2c/2dRC_H_	−17.8	−18.0		−18.7	−19.6	
	2a/2bRC	−17.5	−17.7		−19.4	−20.1	
	2aTS	−11.2	−11.1		−15.8	−16.0	
	2bTS	−11	−11.3		−15.4	−16.9	
	2cTS	−0.1	−6.8		−4.2	−4.9	
	2dTS	31.1	30.0		21.5	19.2	
	2aPC	−31.9	−33.6		−41.5	−42.7	
	2bPC	−42.4	−44.6		−51.8	−50.9	
	2cPC	−44.2	−41.6		−49.0	−54.0	
	2dPC	−42.4	−44.6		−51.8	−54.0	
	2P1	−15.5	−18.1	−22.5	−26.3	−26.2	−37.8
	2P2	−31.3	−34.0	−32.0	−42.1	−44.0	−48.7
^i^C_3_H_7_	3a/3bRC	−20.7	−21.4		−21.9	−22.8	
	3c/3dRC	−15.5	−16.3		−16.9	−17.9	
	3aTS	−11.6	−12.4		−16.9	−17.5	
	3bTS	−9.1	−11.0		−14.1	−16.3	
	3cTS	0.3	−1.0		−4.3	−5.4	
	3dTS	31.5	28.8		21.4	-	
	3aPC	−31.8	−33.8		−41.8	−44.4	
	3bPC	−43.9	−46.9		−54.0	−57.9	
	3cPC	−44.4	−47.3		−48.8	−52.8	
	3dPC	−44.4	−47.3		−54.0	-	
	3P1	−13.9	−16.5	−22.1	−24.8	−28.4	−37.7
	3P2	−31.8	−34.9	−30.3	−42.8	−46.8	−45.9
^t^C_4_H_9_	4a/4bRC	−23.1	−23.3		−24.4	−25.6	
	4c/4dRC	−15.6	−16.1		−17.2	−17.8	
	4aTS	−11.9	−17.6		−17.7	−24.4	
	4bTS	−2.3	-		−7.5	-	
	4cTS	0.8	−4.6		−3.9	−10.8	
	4dTS	39.9	-		28.8	-	
	4aPC	−33.4	−37.8		−43.4	−51.2	
	4bPC	−46.2	-		−56.1	-	
	4cPC	−44.0	−49.1		−49.1	−57.4	
	4dPC	−46.2	-		−56.1	-	
	4P1	−12.6	−18.2	−22.1	−23.9	−37.6	−37.6
	4P2	−31.9	-	−29.5	−43.3	-	−37.8

^a^ (1) Energies with respect to the F^−^ + CH_3_Cl reactants at AE-CCSD(T)/aug-cc-pCVQZ level are from ref. [41]. (2) Energies for F^−^ +C_2_H_5_Cl reaction are obtained at the CCSD(T)-F12b/aug-cc-pVQZ + Δcore level of theory from ref. [16]. (3) Energies for F^−^ + ^i^-C_3_H_7_Cl reaction are calculated at CCSD(T)/aug-cc-pVTZ//MP2/aug-cc-pVDZ level of theory. (4) Energies For F^−^ + ^t^-C_4_H_9_Cl reaction obtained at B97-1/aug-cc-pVDZ level are from ref. [43]. ^b^ (1) Energies of F^−^ + CH_3_I reaction at CCSD(T)-F12b/aug-cc-pVTZ-pp are from ref. [47]. (2) Energies of F^−^ + C_2_H_5_I reaction at CCSD(T)/PP/t level are from ref. [17]. (3) Energies for F^−^ + ^i^-C_3_H_7_I reaction are calculated at CCSD(T)/pp/t//MP2/ECP/d level of theory. (4) Energies For F^−^ + ^t^-C_4_H_9_I reaction obtained at B97-1/aug-cc-pVDZ level are from ref. [43]. ^c^ The reaction enthalpies of reaction at 0 K with ZPE calculated from tabulated reaction enthalpies in ref. [41].

## Data Availability

The data presented in this study are available in Supplementary Material.

## Supplementary Materials

The following supporting information can be downloaded at: https://www.mdpi.com/article/10.3390/molecules28176269/s1, Figure S1: Potential energy curves and stationary points at MP2/ ECP/d level for F- + RI reactions. The energy (in kcal/mol) is relative to F- + CH3I (a) and F- + RI (b) reactants at 0K, and does not include ZPE. For Figure S1b, the black, blue, and pink numbers represent F- + C2H5I, F- + iC3H7I, and F- + tC4H9I reactions, respectively; Figure S2: Stationary point structures of E2 and SN2 pathways for (a) F-+ CH3Y, (b) F-+ C2H5Y, (c) F-+ iC3H7Y, (d) F-+ tC4H9Y(Y = Cl, I) reaction optimized at the MP2/aug-cc-pVDZ(ECP/d) theoretical level. Bond distances are in Å, and black/pink line represents Y = Cl/I.; Figure S3: Interaction energy decomposition according to formula ∆*E_int_* = (∆*E_els_* + ∆*E_XC_* + ∆*E_Pauli_*) + ∆*E_orb_* = ∆*E_steric_* + ∆*E_orb_*. ∆*E_els_* is electrostatic interacton term, ∆*E_XC_* is the change of exchange-correlation energy during complexation process, and ∆*E_Pauli_* is the Pauli repulsion effect between electrons in occupied orbitals of the fragments and is invariably positive. They combine to form steric term ∆*E_steric_* (Dashed line). ∆*E_orb_* is orbital interaction term and represented by short dotted line. The full line represents ∆*E_int_*.
