# Supplementary material for: Effects of Methyl Substitution and Leaving Group on E2/SN2 Competition for Reactions of F− with RY (R = CH3, C2H5, iC3H7, tC4H9; Y = Cl, I)
Source: Molecules. 2023 Aug 27;28(17):6269. doi: 10.3390/molecules28176269 (PMC10488877; doi:10.3390/molecules28176269)
Supplement: Supplementary file 1 [file molecules-28-06269-s001.zip › molecules-2566983-supplementary.pdf]

Effects of Methyl Substitution and Leaving Group on E2/S_N2 Competition for Reactions of F⁻ with RY (R = CH_3, C_2H_5, $^iC_3H_7$, $^tC_4H_9$; Y = Cl, I)

Wenqing Zhen, Siwei Zhao, Gang Fu, Hongyi Wang, Jianmin Sun, Li Yang * and Jiaxu Zhang *

State Key Laboratory of Urban Water Resource and Environment, School of Chemistry and Chemical Engineering, Harbin Institute of Technology, Harbin 150001, China; 20b925102@stu.hit.edu.cn (W.Z.); 18b925114@stu.hit.edu.cn (S.Z.); 20b925088@stu.hit.edu.cn (G.F.); wanghongyi999123@163.com (H.W.); sunjm@hit.edu.cn (J.S.)

* Correspondence: yangli2014@hit.edu.cn (L.Y.), zhjx@hit.edu.cn (J.Z.)

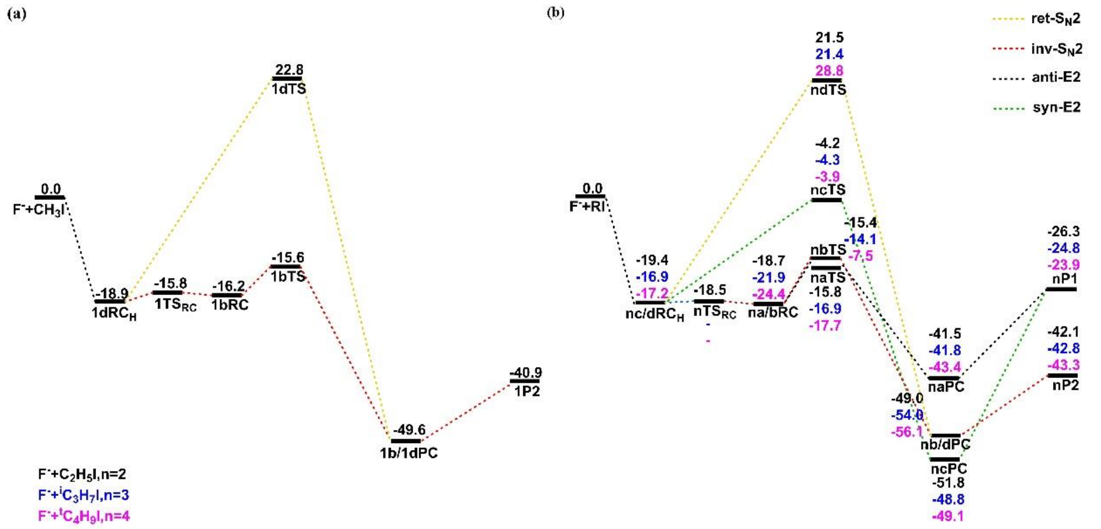

Figure S1. Potential energy curves and stationary points at MP2/ ECP/d level for F⁻ + RI reactions. The energy (in kcal/mol) is relative to F⁻ + CH_3I (a) and F⁻ + RI (b) reactants at 0K, and does not include ZPE. For Figure S1b, the black, blue, and pink numbers represent F⁻ + C_2H_5I, F⁻ + $^iC_3H_7I$, and F⁻ + $^tC_4H_9I$ reactions, respectively.

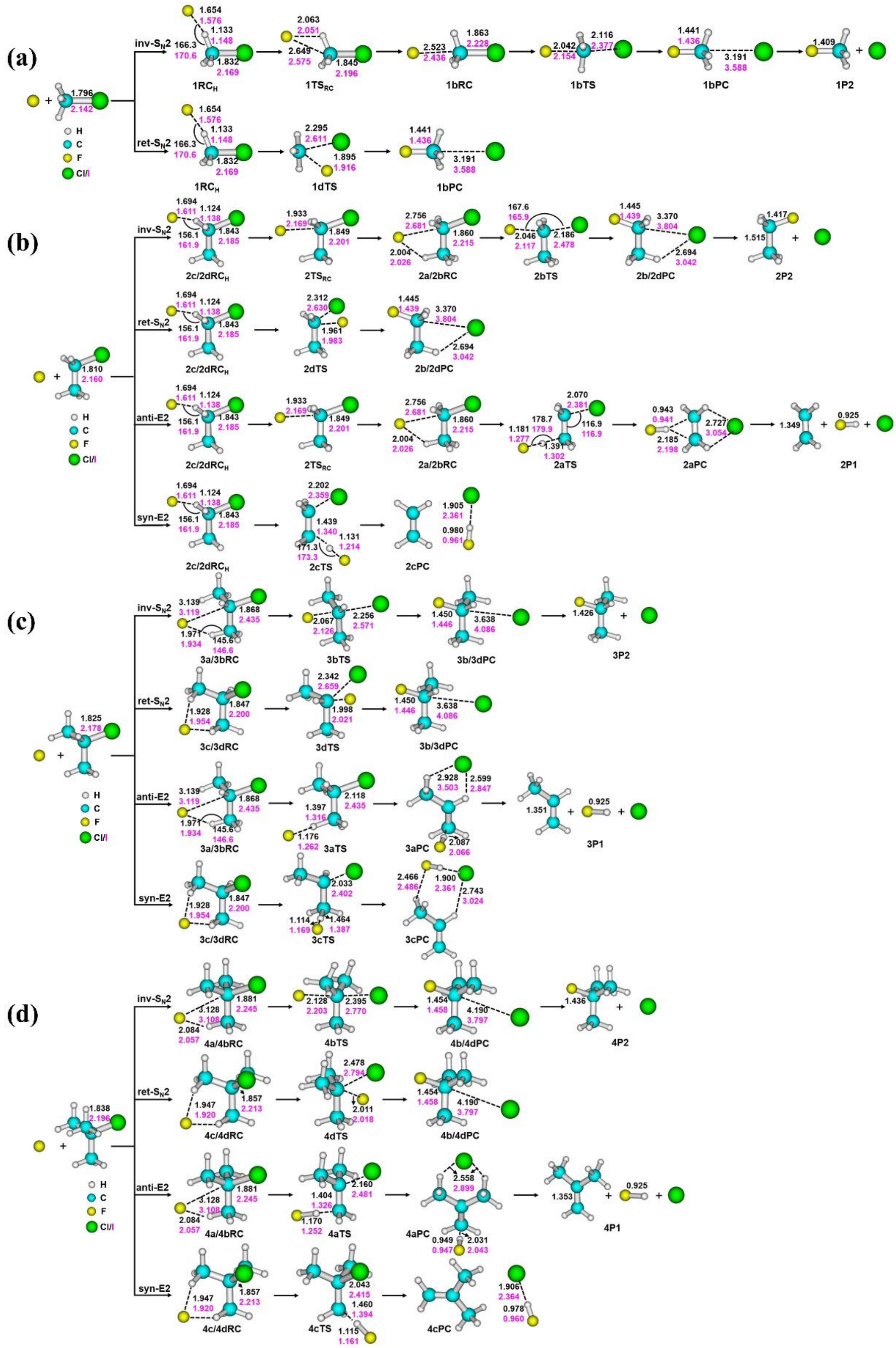

Figure S2. Stationary point structures of E2 and S_N2 pathways for (a) F^-+ CH_3Y, (b) F^-+ C_2H_5Y, (c) F^-+ $^iC_3H_7Y$, (d) F^-+ $^tC_4H_9Y$(Y = Cl, I) reaction optimized at the MP2/aug-cc-pVDZ(ECP/d) theoretical level. Bond distances are in Å, and black/pink line represents Y = Cl / I.

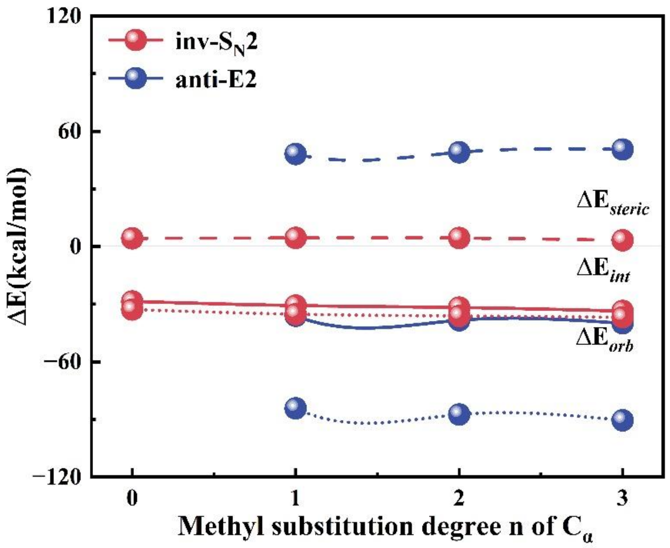

Figure S3. Interaction energy decomposition according to formula $\Delta E_{int} = (\Delta E_{els} + \Delta E_{XC} + \Delta E_{Pauli}) + \Delta E_{orb} = \Delta E_{steric} + \Delta E_{orb}$. ΔE_{els} is electrostatic interacton term, ΔE_{XC} is the change of exchange-correlation energy during complexation process, and ΔE_{Pauli} is the Pauli repulsion effect between electrons in occupied orbitals of the fragments and is invariably positive. They combine to form steric term ΔE_{steric} (Dashed line). ΔE_{orb} is orbital interaction term and represented by short dotted line. The full line represents ΔE_{int}.
